# Analysis methods for measuring passive auditory fNIRS responses generated by a block-design paradigm

**DOI:** 10.1117/1.NPh.8.2.025008

**Published:** 2021-05-22

**Authors:** Robert Luke, Eric Larson, Maureen J. Shader, Hamish Innes-Brown, Lindsey Van Yper, Adrian K. C. Lee, Paul F. Sowman, David McAlpine

**Affiliations:** a Macquarie University, Macquarie University Hearing & Department of Linguistics, Australian Hearing Hub, Sydney, New South Wales, Australia; b The Bionics Institute, Melbourne, Victoria, Australia; c University of Washington, Institute for Learning & Brain Sciences, Seattle, Washington, United States; d The University of Melbourne, Department of Medical Bionics, Melbourne, Victoria, Australia; e Eriksholm Research Centre, Oticon A/S, Snekkersten, Denmark; f University of Washington, Department of Speech & Hearing Sciences and Institute for Learning & Brain Sciences, Seattle, Washington, United States; g Macquarie University, Department of Cognitive Science, Faculty of Medicine, Health and Human Sciences, Sydney, New South Wales, Australia

**Keywords:** auditory responses, block-design paradigm, analysis methods, speech, passive task

## Abstract

**Significance:** Functional near-infrared spectroscopy (fNIRS) is an increasingly popular tool in auditory research, but the range of analysis procedures employed across studies may complicate the interpretation of data.

**Aim:** We aim to assess the impact of different analysis procedures on the morphology, detection, and lateralization of auditory responses in fNIRS. Specifically, we determine whether averaging or generalized linear model (GLM)-based analysis generates different experimental conclusions when applied to a block-protocol design. The impact of parameter selection of GLMs on detecting auditory-evoked responses was also quantified.

**Approach:** 17 listeners were exposed to three commonly employed auditory stimuli: noise, speech, and silence. A block design, comprising sounds of 5 s duration and 10 to 20 s silent intervals, was employed.

**Results:** Both analysis procedures generated similar response morphologies and amplitude estimates, and both indicated that responses to speech were significantly greater than to noise or silence. Neither approach indicated a significant effect of brain hemisphere on responses to speech. Methods to correct for systemic hemodynamic responses using short channels improved detection at the individual level.

**Conclusions:** Consistent with theoretical considerations, simulations, and other experimental domains, GLM and averaging analyses generate the same group-level experimental conclusions. We release this dataset publicly for use in future development and optimization of algorithms.

## 1 Introduction

Functional near-infrared spectroscopy (fNIRS) is an increasingly popular technique^1^ employed to investigate auditory-cortical function, and provides a unique set of qualities that make it ideal for auditory research. fNIRS devices are typically very quiet compared with functional magnetic resonance imaging (fMRI) with which it shares a similar biologically generated signal. fNIRS is unaffected by electrical or magnetic interference from hearing devices such as cochlear implants or hearing aids, all of which are either contra-indicated or generate large artifacts in fMRI and in electro- and magneto-encephalography (EEG and MEG, respectively). fNIRS devices are generally relatively portable and do not require participants or patients to be isolated in a shielded chamber or to have their head-position fixed, making it well suited for use in low- or non-compliant groups, including children, the elderly, and the cognitively impaired. Therefore, it provides an ideal imaging modality for clinical applications.

fNIRS has been used to investigate a variety of auditory research questions and applications. A primary use has been the investigation of cortical processing of physical qualities of sound, such as intensity, amplitude and frequency modulations, and auditory-spatial cues.^2^^–^^4^ fNIRS has also been employed to evaluate the perceptual qualities of speech and listening effort and language development in normal-hearing and hearing-impaired populations.^5^^–^^14^ Research questions relating to the development of auditory cortical function^15^ and cortical reorganization following impaired sensory input and subsequent rehabilitation^16^^,^^17^ have been investigated using fNIRS, as have outcomes related to cochlear implantation^5^ and auditory pathologies such as tinnitus.^18^^,^^19^

Of particular clinical relevance within the auditory research community is the field of objective measures. Objective measures utilize passive experimental designs—in which the participant is not required to perform any tasks throughout the measurement—and are routinely utilized to evaluate hearing performance in populations who are unable to provide reliable behavioral responses. Specifically, EEG-based objective measures are used clinically to evaluate frequency-specific hearing thresholds in newborn children;^20^^–^^22^ in research environments to evaluate auditory function including modulation sensitivity,^23^ binaural sensitivity,^24^ speech reception,^25^ and auditory pathway development;^26^ and to evaluate the interface between cochlear implant electrodes and neural tissue.^27^ Due to its favorable qualities for studying auditory function, fNIRS has been indicated as a promising neuroimaging technique for auditory objective measures. However, the existing literature evaluating fNIRS analysis methodologies and artifact rejection techniques has primarily focused on active experimental designs in which the participant performs a task. As such, in this paper, we investigate analysis procedures when applied to a passive auditory task, as would be utilized in auditory objective measures.

Despite the utility of fNIRS for investigating auditory function, however, relative to other neuroimaging modalities such as fMRI, EEG, and MEG, fNIRS has been employed only recently by hearing scientists, and considerable variability exists in the experimental designs and analysis techniques used by different researchers. This variability can make it difficult to interpret data sets or to replicate or compare findings across studies or between research teams. The experimental designs most commonly employed by auditory fNIRS researchers are block and event-related designs. When selecting an experimental design, researchers must consider a range of factors, including the statistical power of the protocol, the duration of the experiment, and whether the design provides the flexibility to study the effect of interest.^28^^–^^31^ For example, an event-related design may enable an investigator to examine the response to individual words in an ongoing sentence, something not possible when employing a block design. Here, we compare two common analysis procedures that can be applied in experiments employing a block design with a passive-auditory paradigm.

Block-design experiments present a single stimulus continuously for an extended time interval (e.g. 5 s), followed by an inter-stimulus interval (i.e., in which no stimulus is presented) of sufficient duration for the hemodynamic response to return to an approximate basal level.^32^^,^^33^^,^^34^ Although commonly employed, no consensus exists as to the most appropriate analysis procedures for this type of experimental design; new algorithms and procedures are regularly published without cross-validation or theoretical consideration.

Analysis procedures for block designs typically lie in one of two categories: averaging analysis, in which the fNIRS measurement is segmented and averaged relative to the onset of the stimulus,^35^ and general linear model (GLM) analysis, in which one or more model hemodynamic responses are fitted to the entirety of the measured fNIRS signal [Ref. 36; for a recent overview in the context of fNIRS, see Ref. 37]. The signal-averaging approach assumes that the noise component of the measured fNIRS signal is a random process with zero mean and is unrelated to the biological signal of interest. In contrast, the GLM is capable of accounting for a more complex model of signal noise.^38^ Although for non-overlapping responses such as those assumed in a block design, the GLM model is reduced to a block average, suggesting that both analyses should generate similar outcomes;^39^^,^^40^ due to the statistical properties of the fNIRS signal, GLM analysis may be a more appropriate method with which to analyze fNIRS data.^37^ These two analysis methods have been described and evaluated for different fNIRS analysis parameters in computer simulations and behavioral motor experiments,^39^^,^^41^ but a direct comparison has yet to be made for research investigating audition.

In general, auditory-cortical responses in fNIRS have been shown to be reliable at a group level.^42^ Many investigations of auditory-cortical function target relatively deep (relative to the skull) cortical regions such as Heschl’s gyrus, of which a typical fNIRS device might generate <1% specificity.^43^ This low specificity makes individual-level measurements unreliable, largely due to the poor signal-to-noise ratio; the measured stimulus-evoked hemodynamic response is small compared with all other sources of bio-generated changes in the fNIRS signal. This challenge has motivated the need for a comparison of averaging and GLM analysis specifically for auditory fNIRS signals to understand the influence of analysis choices when analyzing such a small signal-of-interest. Here, we investigate whether averaging and GLM analysis applied to the same dataset generate results that support the same experimental conclusions.

This study focuses specifically on a passive experimental paradigm. In addition to being of important clinical relevance to the auditory community, this type of study provides several benefits when interpreting the resulting neuroimaging data. First, a passive task reduces the contribution of systemic components (changes in the measured fNIRS signal that are not due to the effect of neurovascular coupling) to the fNIRS signal. Active tasks such as mental arithmetic, inner speech, and arm movements can evoke task-related changes in respiration and mean arterial blood pressure,^44^ which may mask the neural effect of interest. Second, limiting the actions of the participant reduces the number of confounding neural processes that must be disentangled to study the effect of interest. For example, if participants are required to provide a verbal response, the act of speech production will increase neural activity in the superior temporal gyrus,^45^ and even if the verbal response is delayed relative to the event of interest, the act of speech planning may also induce additional neural activity.^46^ A common alternative task in auditory experiments is to have the participant press a button; however, this is undesirable as motor movements may modulate auditory cortical activity.^47^ In studies that require active participation from the listeners, modifications to experimental design, such as jittering the response window and additional signal processing, can be used to mitigate undesired systemic and neural components.

Due to the statistical properties of the noise within fNIRS signals, GLM-style analysis has been suggested to be a more appropriate method with which to analyze fNIRS data.^37^ As such, we also investigated the influence of the parameters employed in GLM analysis on the true and false detection rates of sound-generated fNIRS responses. Of particular importance in fNIRS experiments is the separation (and possible reduction) of systemic contributions to the measured signal when estimating neural responses.^44^ This has particular relevance for auditory experiments as systemic components of fNIRS measurements have been shown to be related to the characteristics of acoustic stimuli.^48^

Many approaches have been proposed to remove the influence of systemic components on the estimation of the neural response.^49^^–^^53^ Most use specialized channels designed to measure the systemic response only, and not neural activity. These channels typically have a source-detector separation of <1  cm and are often referred to as “short” channels. Recently, Santosa et al.^51^ concluded that including short-channel information as a regressor of no interest within a GLM analysis resulted in the most accurate estimation of the underlying neural response compared with spatial and temporal filtering, regression, and component analysis.

We, therefore, investigated the effect of including information from short channels on the detection of auditory fNIRS responses. Algorithms that remove systemic components have previously been evaluated and contrasted,^51^^–^^53^ but we apply these methods specifically in the context of a passive task with two commonly used auditory stimuli: speech and bandpass noise.

Speech is the primary mode for auditory communication and is therefore widely employed in auditory experiments. Noise signals are often used to investigate basic auditory processing as the statistical properties of the signal can be precisely controlled. These two stimuli are often contrasted to investigate language-specific processing or combined to investigate speech processing in challenging listening environments. Both stimuli can hold an infinite number of forms; speech may contain prosodic cues or be spectrally degraded, and noise may comprise different frequency ranges, contain modulations in amplitude or frequency, or transition over time. Here, we employed two different stimuli: speech comprising three concatenated sentences in quiet and a 400-Hz band of noise centered at 500 Hz.

We first describe the methods used to produce and present stimuli and to generate data. We then undertake qualitative analysis examining the morphology of fNIRS responses to auditory stimuli using averaging and GLM analyses, and we assess the influence of different analysis parameters on the detection of auditory fNIRS responses and on the rate of false positives. Finally, we investigate whether the averaging and GLM approaches provide similar experimental conclusions when applied to the same dataset. Both approaches were used to investigate two common questions in auditory neuroscience. First, do two different stimulus conditions generate a different response amplitude? Second, are cortical-hemispheric differences apparent in evoked responses?

One challenge when developing an experimental protocol for fNIRS is to understand the effects of different analysis choices and to optimize the signal-processing procedure. Further, it is important not to optimize a specific analysis pipeline using the same data from which scientific conclusions will be drawn.^54^ The dataset that we report here will be released publicly to assist in the development of future auditory fNIRS pipelines and algorithm development. In a similar vein, we note that we are not endeavoring to generate scientific conclusions concerning the relative cortical processing of speech and noise. Rather, our intention is to provide an understanding of the choice of parameters on conclusions reached by statistical analysis of auditory-generated fNIRS responses generated using averaging and GLM techniques.

## 2 Methods

### 2.1 Experimental Design

Seventeen participants volunteered for this project. All participants indicated no history of hearing concerns. Participants were aged between 22 and 40 years. Data were collected under the Macquarie University Ethics Application Reference 52020640814625.

**Fig. 1:**
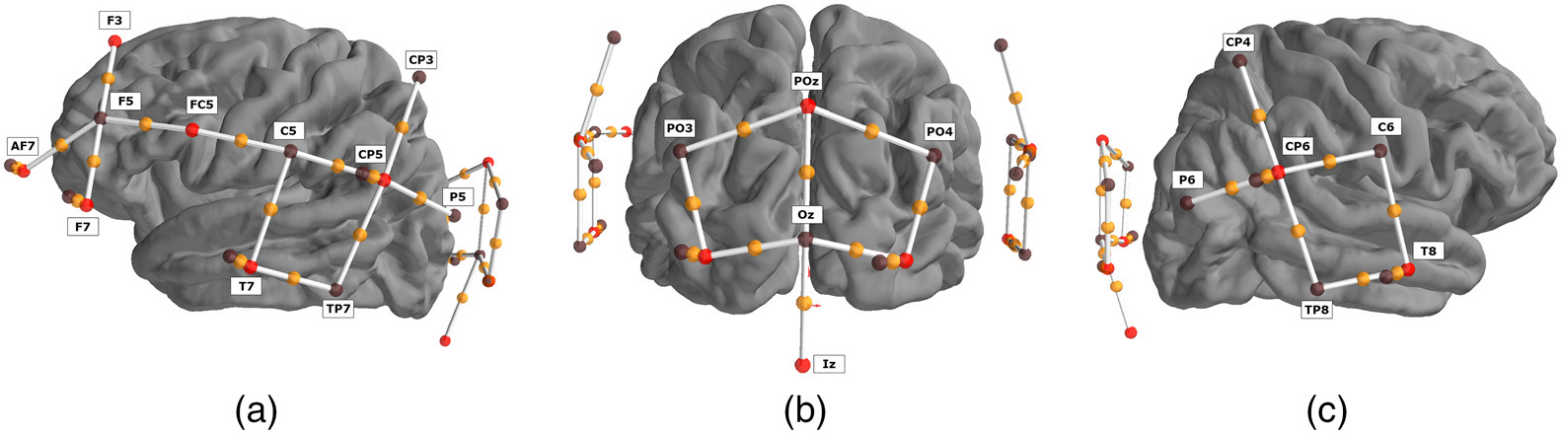
Location of sources and detectors. Four ROI were created to cover the left IFG, the left and right STG, and the occipital lobe. Sources are shown as red dots, detectors are shown as black dots, and channels are shown as white lines with an orange dot representing the midpoint. The montage is shown from the (a) left; (b) back; and (c) right views of the brain.

Participants were seated in a sound-attenuating booth in a comfortable chair for the duration of the experiment, which lasted approximately 25 min. Participants were instructed not to pay attention to the sounds and were offered the choice of watching a silent, subtitled film during the experiment; seven participants accepted this option. NIRS data were recorded using a NIRx NIRScoutX device with APD detectors. The data were saved to disk with a sample rate of 5.2 Hz. 12 source channels and 12 detector channels were employed in the fNIRS optode-cap configuration, with eight additional short detectors distributed across the head. Sources were placed at the positions AF7, F3, F7, FC5, T7, CP5, O1, POz, O2, Iz, CP6, and T8. Detectors were placed at the positions F5, C5, TP7, CP3, P5, PO3, P04, Oz, P6, CP4, TP8, and C6. Short detectors were placed at AF7, F7, T7, CP5, O1, O2, CP6, and T8 (Fig. 1). These optodes were selected to target four regions of interest (ROI) using the fOLD toolbox,^43^ including the left inferior frontal gyrus (IFG), which consisted of channel pairs AF7-F5, F3-F5, F7-F5, and FC5-F5; the left and right superior temporal gyri (STG), which consisted of channel pairs T7-C5, T7-TP7, CP5-C5, CP5-TP7, CP5-CP3, and CP5-P5 and CP6-P6, CP6-TP8, CP6-CP4, CP6-C6, T8-TP8, and T8-C6, respectively; and the occipital lobe, which consisted of channel pairs O1-P03, O1-Oz, POz-PO3, POz-Oz, POz-PO4, Iz-Oz, O2-Oz, and O2-PO4. This montage resulted in a total of 24 source-detector pairs (channels). The left IFG is indicated in speech and language processing, while the STG is indicated in auditory processing. The occipital lobe is indicated in visual processing and as a possible additional site for speech processing, particularly in cross-modal plasticity studies, but this region was not expected to show significant responses in the current study.

Participants listened to auditory stimuli presented diotically (i.e., the same sound to both ears) via Etymotic Research ER-2 insert-phones connected to an RME Fireface UCX soundcard (16 bits, 44.1 kHz sampling rate). Speech was presented at 80 dB SPL and noise (separately) at 85 dB SPL. Stimuli were calibrated to a Casella Cel-110/2 sound source using a Norsonic sound-level meter (Norsonic SA, Norway) and an ear simulator (RA0045 G.R.A.S., Denmark).

Participants were exposed to three stimulus conditions: speech, noise, and silence. The speech stimulus consisted of three concatenated sentences from the AusTIN speech corpus^55^ with a total duration of 5.25 s. The noise stimulus consisted of a uniform distribution of frequency content between 300 and 700 Hz and was of 5 s duration. Five seconds of silence was used as the control condition. Stimuli were presented in a random order with an inter-stimulus interval (defined as the time between the offset of one stimulus to the onset of the following stimulus) selected randomly for each trial from a uniform distribution in the range of 10 to 20 s. Twenty trials were presented for each condition, resulting in a total of 60 trials per participant. This is a relatively large number of trials compared with typical group-level fNIRS auditory studies and was selected to ensure an accurate estimation of the response morphology. For both averaging and GLM analysis, more trials will result in a more powerful estimation of the hemodynamic response, and the number of trials must be balanced against the increase in experiment test time.

### 2.2 Analysis

All analyses were performed using MNE (version 0.21.2)^56^^,^^57^ and MNE-NIRS (version 0.0.1),^58^ which makes extensive use of the Nilearn package (version 0.70)^59^ for GLM analysis. First, a qualitative analysis was performed to understand the morphology of the measured signal, followed by a quantitative analysis to evaluate the influence of different parameters on the detection of auditory responses. Finally, both the averaging and GLM analysis techniques were used to compare the response amplitude to speech versus noise and for relative activation in the left versus right cortical hemispheres. All analyses were applied to the same dataset described in Sec. 2.1. This analysis predominantly focuses on the ROI covering the STG. This brain region is expected to be activated by both speech and noise stimuli, whereas expectations of how the occipital and inferior frontal regions will respond to these stimuli is still an active area of research^9^^,^^14^^,^^60^ and is beyond the scope of this article

#### 2.2.1 Morphology of auditory responses

Hemodynamic responses vary with location on the scalp and experimental conditions.^61^^,^^62^ As such, morphology of fNIRS responses to speech and noise stimuli was investigated qualitatively using two independent procedures. The first procedure was an averaging style analysis, and the second was a finite impulse response (FIR) GLM approach. Each analysis was performed on each of the three experimental conditions.

##### Averaging analysis

The averaging analysis consisted of several steps, starting with downsampling the data to 3 Hz and conversion to optical density. The scalp-coupling index^8^ was calculated for each channel between 0.7 and 1.45 Hz (corresponding to heart rates between 42 and 87 beats per second, consistent with the range expected for a relaxed adult). Channels with a scalp-coupling-index value below 0.8 were removed, resulting in different numbers of channels being removed for different participants. Data from each channel were then further cleaned by applying temporal-derivative distribution repair^63^ and short-channel regression based on the nearest short channel.^50^^,^^52^ Briefly, this approach to short-channel regression subtracts a scaled version of the signal obtained from the nearest short channel from the signal obtained from the long channel. The modified Beer–Lambert law was then applied, with a partial pathlength factor of 0.1,^64^^–^^66^ converting the optical-density measurements to changes in hemoglobin concentration. Next, channels with source-detector separations outside the range of 20 to 40 mm were excluded, followed by application of the signal-improvement algorithm based on the negative correlation between oxygenated and deoxygenated hemoglobin dynamics.^67^ A bandpass filter was then applied between 0.01 and 0.7 Hz with a transition bandwidth of 0.005 and 0.3 Hz for the low- and high-pass edges, respectively. The data were cut into epochs from 3 s before stimulus onset to 14 s after (ensuring no conflict with the onset of the next stimulus presentation, which may occur as early as 15 s after the previous stimulus onset), and a linear detrend was applied to each epoch. Epochs with a peak-to-peak difference in any channel exceeding 100  μM were then excluded. The average response per participant for each channel and for each condition was exported. Then the channels within a region of interest were averaged to create an ROI average waveform for each participant. Due to the channel removal based on the scalp coupling index above, there may be a different number of channels included within this average for each participant and ROI. To summarize the group-level averaging analysis results, the ROI average waveforms were used to create a time series visualization displaying the average signal across participants and a bootstrapped 95% confidence band around the mean for each condition and ROI.

##### Finite impulse response model analysis

In a second, independent analysis, data was entered into a GLM analysis using a deconvolution FIR model. This method makes no assumptions as to the shape of the hemodynamic response. Instead, a series of impulses following the onset of the stimulus are used as regressors to model the neural response. The morphology of the response can then be estimated by summing all of the FIR components after multiplication by each component’s weight as estimated by the GLM. See Huppert^37^ and Santosa et al.^66^ for a summary of FIR and canonical approaches within the fNIRS context.

Prior to the GLM analysis, data were downsampled to 1 Hz and then converted to optical density. A lower sample rate was employed as the scalp-coupling index was not computed, and therefore, higher frequencies were not required. Next, channels with a source-detector separation outside the range of 20 to 40 mm were excluded, and the modified Beer–Lambert law was applied to the data, as performed in the averaging analysis. A GLM was then applied using a FIR model with 14 components (i.e., 14 s); this number of components was selected to ensure parity with the epoching-window approach employed in the averaging analysis. Channels were then combined into a ROI by averaging the estimates with an inverse weighting by the standard error of the GLM fit. The individual-level FIR results were then entered into a linear mixed-effects (LME) model to extract the effect of FIR delay, condition, and chromophore, while accounting for the random effect of subject. Santosa et al.^66^ provides for a description of these second-level statistical models.

#### 2.2.2 Canonical model analysis: effect of parameters on response detection

Next, the effect of several analysis parameters on the detection rate for auditory responses was investigated. In contrast to the FIR approach (Sec. 2.2.1), this analysis used a predefined canonical model of the evoked hemodynamic response function (HRF), specifically the canonical SPM HRF, which is generated from a linear combination of two Gamma functions.^68^ The effect of sampling rate, correction for systemic responses, and boxcar duration on the true- and false-positive detection rates was explored. For simplicity, we visualized only the data for oxyhemoglobin, and not deoxyhemoglobin, as the effects of different parameters were similar for both. Only responses from optodes placed over the STG were analyzed.

Specific analysis parameters were varied in this section, but each analysis consisted of the same general procedure—re-sampling the data, followed by conversion to optical density and hemoglobin concentration. Next, channels with source-detector separation outside the 20- to 40-mm range were excluded, as were any channels outside the STG ROI. A design matrix was then constructed by creating a boxcar function based on the trigger timing and convolving this with the SPM HRF. Drift factors up to 0.01 Hz were also included in the GLM as a regressor to model the low-frequency oscillations in the data. An alternative approach may be to prefilter the data.^69^ A GLM was performed on the data with this design matrix, including the use of a fourth-order auto-regressive noise model, generating channel-level data that were used to construct a receiver operating characteristic (ROC) curve. Channel-level data were then combined into a ROI by applying a weighted-average procedure to the estimated coefficients, in which each channel was weighted by the inverse of the standard error of the GLM fit for individual channels. This procedure was termed the “no correction” analysis.

A false positive was defined as a response detected in the (control) condition of silence. A true positive was defined as a response detected in the speech and noise conditions. Using these definitions, a ROC curve was generated by varying the threshold applied to the p value to determine detection of a response (either a false positive or a true positive). The ROC was defined for each analysis procedure, and the area under the curve was extracted to quantify the analysis performance at an individual level. We also extracted the true-positive rate (TPR) resulting from a false-positive rate (FPR) of 5%, as commonly employed in clinical studies.

To analyze the effect of different choices of processing, several modifications were made to the procedure outlined above. Different short-channel approaches were applied to correct for systemic response, including adding the mean of the short channels as a regressor to the GLM, adding the individual short channels as regressors to the GLM, and adding the principal components (PCs) of the short channels as regressors to the GLM (adding either a subset or all of the components was investigated). These procedures were termed the “systemic corrected” analysis. Similarly, the effect of sample rate was investigated by downsampling the raw signal using different rates.

#### 2.2.3 Comparison of conditions and response lateralization

Finally, a group-level analysis was performed to determine if the averaging and GLM analyses provided the same conclusion to two research questions. First, is there a difference in response amplitude between the speech and noise stimuli? Second, is there a hemispheric difference in the response to speech stimuli? We focus on group-level analysis as this has been demonstrated to be reliable in auditory fNIRS experiments.^42^ We also investigate whether including the approach to correcting for the systemic response correction deemed most effective (see Sec. 2.3) modifies the experimental conclusions.

##### Averaging analysis

For the averaging analysis, the same approach was made as in Sec. 2.2.1, after which, the mean value between 5 and 7 s of the average waveform for each participant was exported for analysis by statistical testing. fNIRS responses to relatively short sentences have been shown to peak within this time range.^12^

##### Canonical model analysis

For the canonical-model GLM analysis, two procedures were used: the no correction approach and the systemic corrected approach, the latter of which included all PCs as regressors in the GLM to compensate for systemic responses. Both analyses (as described in Sec. 2.2.2) downsampled the data to 0.6 Hz and used a 3 s duration for the boxcar function.

##### Statistical analysis

To summarize the dataset, results from the systemic corrected approach were entered into a LME model that accounted for condition, ROI, and chromophore with the participant as a random variable. In Roger–Wilkinson notation, this would be described as β∼−1 + Condition:ROI:Chroma + (1|ID).

For each of the three analyses described above (averaging, GLM No Correction, GLM Systemic Corrected), a response estimate was exported for each participant, each condition, and each ROI. These data were then used to address two issues. First, using all channels over both left and right STG as a single ROI, a LME model was used to determine if the response to speech was different from that to noise. The participant was included as a random effect. In Roger–Wilkinson notation, this is described as β∼Condition+(1|ID). Second, a LME model was used to determine if the left STG shows a different response amplitude than the right in the speech condition, described as β∼ROI+(1|ID). Two separate models were applied, one for the oxyhemoglobin values and one for the deoxyhemoglobin values.

## 3 Results and Discussion

**Fig. 2:**
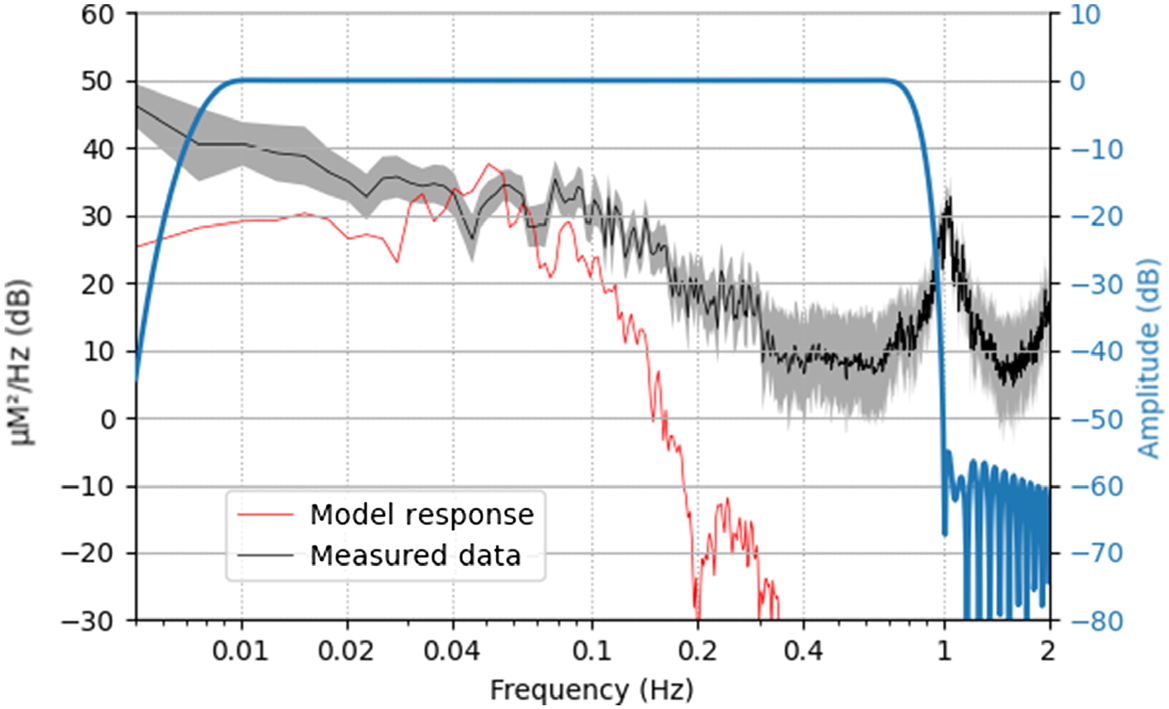
Summary of frequency information. The frequency content of the expected neural response based on trigger information and model HRF is shown in red (arbitrary scaling). The applied filter is shown in blue. Raw data from an example file are shown in black, with the solid line indicating the mean value across all channels and the shading representing 95% confidence intervals across channels. It is worth noting that the filter retains most of the experimental frequency content while removing high-frequency heart rate content (around 1 Hz) and low frequency content in the measured data.

To ensure that the bandpass filter applied in the averaging procedure was parameterized correctly, as to remove unwanted components of the measurements and retain the frequency content of interest, the spectrum of the raw fNIRS data extracted from an example data file is plotted along with the expected hemodynamic response (Fig. 2). The spectral content of the model boxcar function of the experiment convolved with a model neural response (Fig. 2, red curve) indicates that the majority of the signal content is around 0.05 Hz, consistent with the average presentation rate of the experiment. The spectral content of an example measurement (Fig. 2, black curve) indicates a clear signal generated by the systemic pulse rate of around 1 Hz. The filter-frequency response (Fig. 2, blue) clearly retains the peak of the expected response, but it excludes the low-frequency drift and high-frequency (pulse-rate) components.

### 3.1 Morphology of fNIRS Responses to Speech and Noise

Two approaches were applied to investigate the morphology of responses to auditory stimuli in each ROI. Here, we provide a qualitative description of morphology.

#### 3.1.1 Averaging analysis

**Fig. 3:**
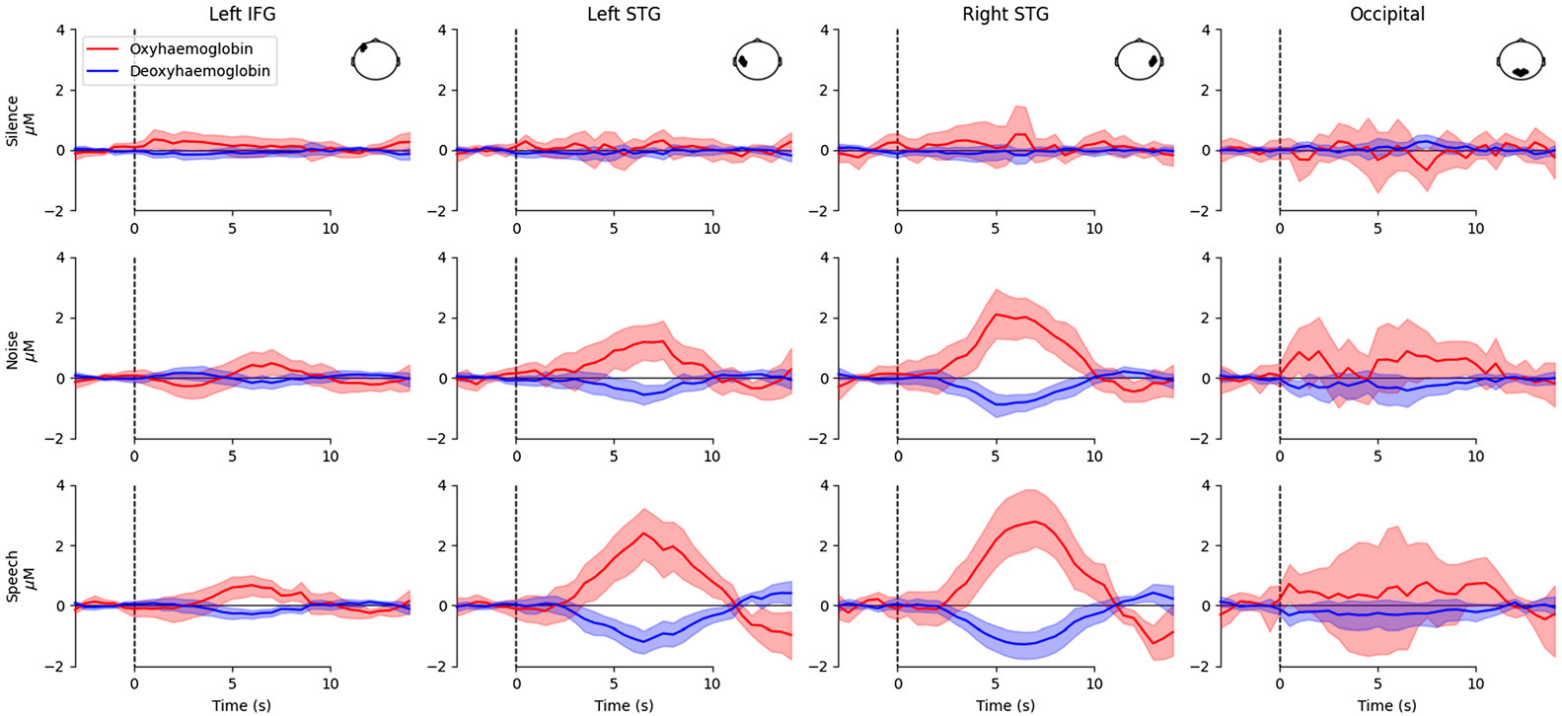
Morphology of auditory fNIRS responses using the averaging approach for all ROI and conditions. Each column represents a different region of interest as illustrated in the top-down head view inset. Each row represents a different stimulus condition. Red represents oxyhemoglobin, and blue represents deoxyhemoglobin. Shaded lines indicate 95% confidence intervals. Responses were observed over the left and right STG for both speech and noise conditions, but not for silence.

To summarize the group-level averaging analysis results, a time series visualizes the average signal across participants and a bootstrapped 95% confidence band around the mean for each condition and ROI (Fig. 3). Responses were observed in the STG regions for both noise and speech stimuli, but not for the silent conditions. For the silence condition, relatively flat measurements were observed over the entire waveform in all ROIs. For both speech and noise conditions, the largest responses were measured from optodes placed over the left and right STG. These responses show a canonical hemodynamic response, with a peak response around 5 to 7 s after stimulus onset, consistent with the duration of the stimulus.^12^ As such, only channels over the left and right STG were used subsequently to quantify response morphology.

#### 3.1.2 Finite impulse response model analysis

**Fig. 4:**
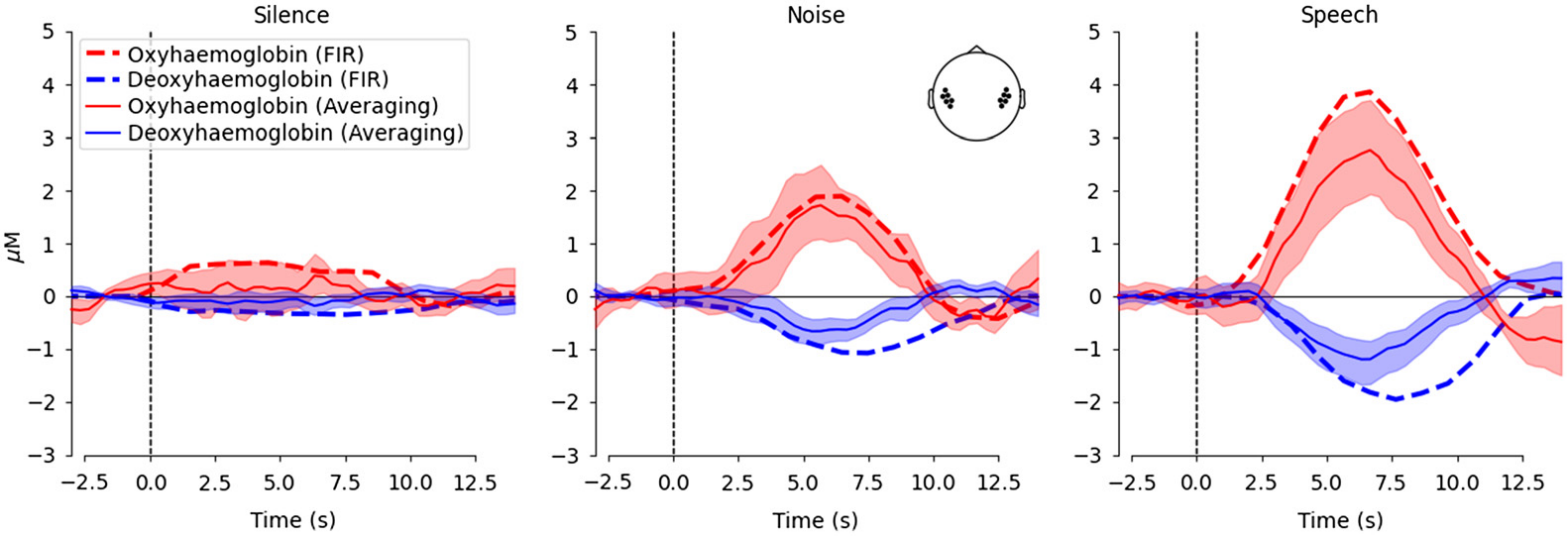
Morphology of auditory fNIRS responses over the STG. Each column represents a different stimulus condition. Responses are illustrated for both oxy- and deoxyhemoglobin in red and blue, respectively. The shaded areas and solid line represent the mean and 95% confidence intervals for the averaging approach. The dashed lines illustrate the estimates for the FIR GLM approach. It is worth noting that the averaging and FIR GLM fits are quite similar, except for a larger estimate for the FIR approach in the speech condition.

A FIR GLM analysis was also used to examine the morphology of the hemodynamic response, using only optodes situated over the STG. A comparison of the estimated response morphology using the averaging and the FIR (GLM) techniques (Fig. 4) indicates broad agreement between the methods with regard to the timing and amplitude of hemodynamic responses, although the FIR approach generates an estimate of the response to speech greater than that suggested by averaging. The morphology of the responses demonstrated in Figs. 3 and 4 are specific to the stimuli used in this study, and response morphology may vary in a nonlinear fashion for stimuli of different content or duration^70^ and for study populations with different ages.^71^ As such, it is important to report the response morphology and verify that an appropriate canonical model is fitted to the data for any GLM analysis.

### 3.2 Canonical Model Analysis: Effect of Parameters on Response Detection

**Fig. 5:**
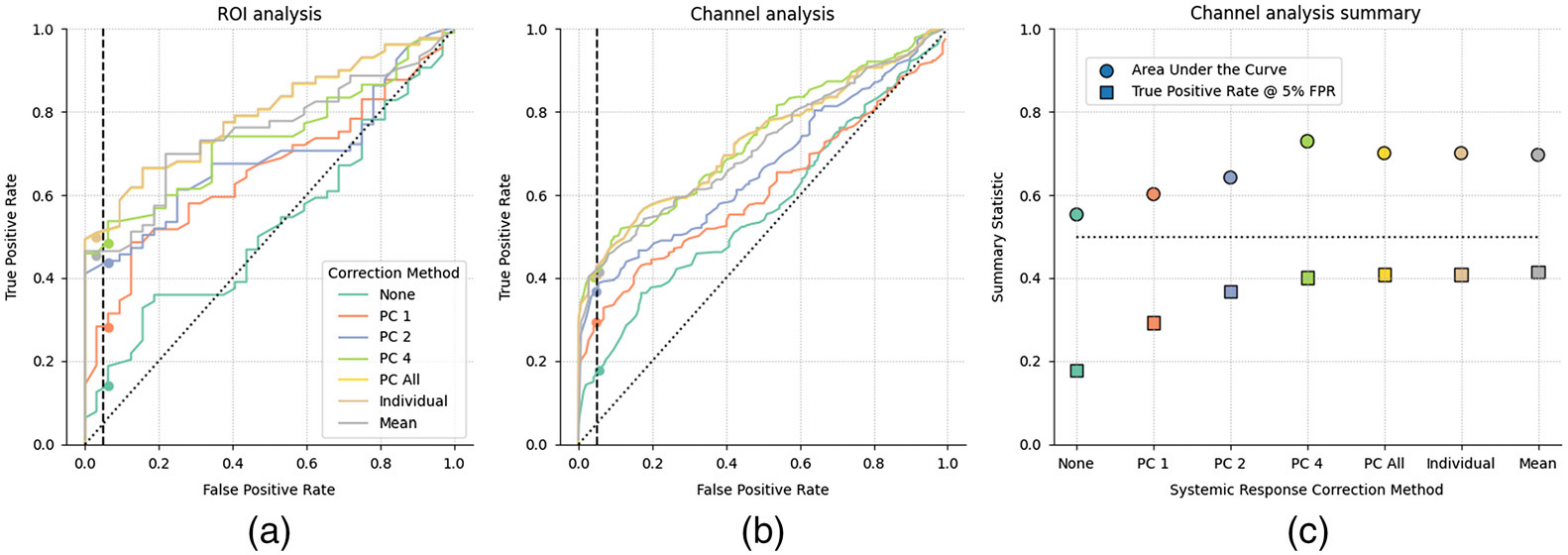
The effect of systemic response correction on auditory fNIRS response estimates. (a) ROC curves for the STG region of interest and (b) individual channels over the STG. (c) Summary statistics from the individual channel ROC with area under the curve (circle) and TPR at 5% F (square) metrics for each method. Analysis with no systemic correction is included as a reference (green), analysis with 1, 2, 4, or all PC of the short channels as regressors in the GLM is shown (orange, blue, light green, yellow, respectively), and all short channels included as individual regressors (brown) or averaged per chromophore (gray). Note that all systemic response correction approaches provide improved detection over no correction. Including all PCs, the mean of the short channels, or all individual channels provides best auditory response detection.

We next examined the effect of different analysis parameters on the detection of responses in individual participants. ROC curves for both ROIs [Fig. 5(a)] and individual channels [Fig. 5(b)] indicate that ROIs show greater sensitivity to true positives than individual channels, likely due to noisy channels being inversely weighted. Subsequently, we focus on the channel-level results [Fig. 5(c)].

Two summary metrics extracted from the ROC curves are reported. First is the traditional area under the curve (AUC) measure. A larger value indicates better performance across the entire range of false-positive values. Also reported is the TPR occurring at the 5% false-positive rate (FPR). We chose to focus on the metric at 5% FPR, as opposed to the AUC metric, because this tends to be more relevant for clinical purposes.^72^^,^^73^ Many of the differences in the ROC occur at a high FPR at and above 50%; however, this FPR would be considered unacceptable in a clinical setting.

#### 3.2.1 Effect of short channel regression on detection of auditory responses

We first examined the effect of different short-channel based methods on reducing systemic responses from the estimated neural responses. The effect of adding different representations of the short channels as regressors in the GLM is explored. These representations include a limited number of PCs, all PCs, the individual short channels, or the mean of the short channels per each chromophore.

Without short-channel correction, responses were detected in <20% of individual channel measurements for an FPR of 5%. As expected, applying the short-channel method to remove systemic components resulted in a substantial improvement in the detection rate.^41^^,^^51^^–^^53^ Although it is common to use just the first or second PCs as regressors,^2^ we observed that including all components resulted in the best performance, consistent with Huppert.^74^ Including either all PCA components, the mean, or all individual short channels simplifies analysis as these approaches do not require a specific selection criterion, making them easy to implement, describe, and replicate.

We also observed that including all of the individual short channels or the mean of the short channels as regressors, instead of the PCs, also results in detection rates equally as good as the PCA approach. Recent reports utilizing active tasks has suggested that including all PCs is the most effective method for compensating for systemic components;^74^ however, this was not the case in the current dataset. This suggests that meaningful comparisons can be made across studies that include different short-channel regressor methods in their GLM analysis in passive-auditory studies on an individual-level analysis. For the remainder of this study, we selected to use all the PCs as regressors for subsequent analysis as this is suggested to be the most effective method for compensating for systemic components in the estimation of neural responses.^74^

#### 3.2.2 Effect of sample rate on the detection of auditory responses

**Fig. 6:**
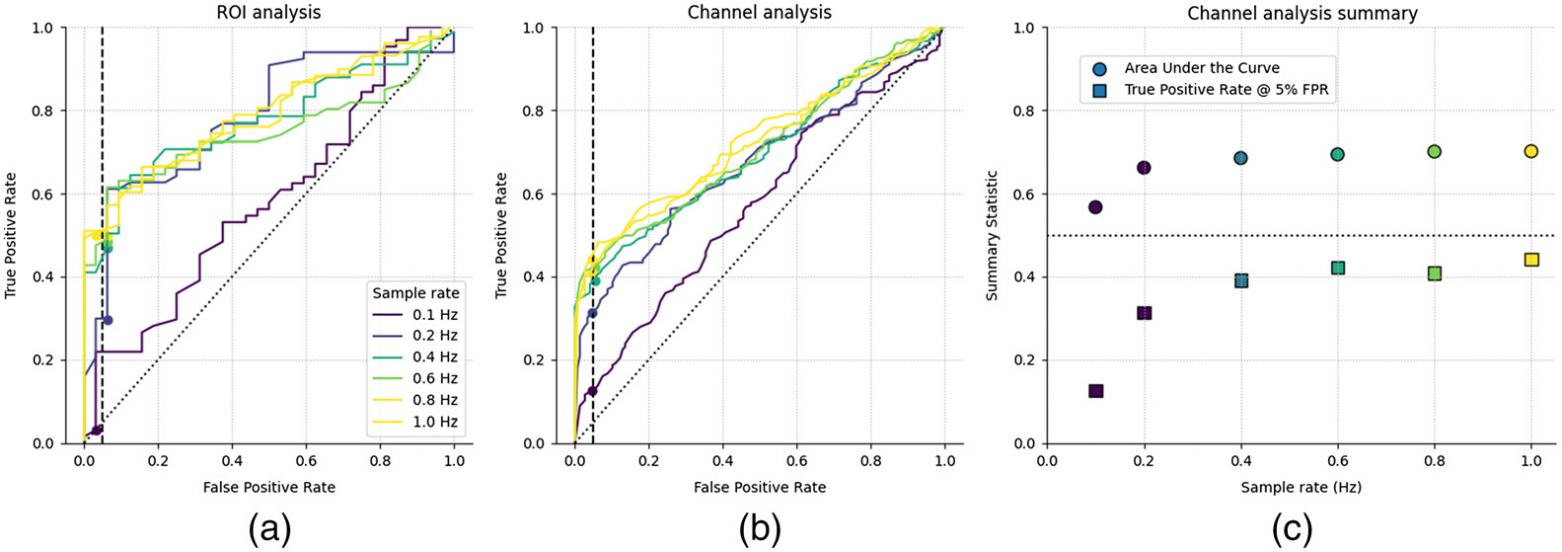
The effect of sample rate on auditory fNIRS response estimates. (a) ROC curves for the STG region of interest and (b) individual channels over the STG. (c) Summary statistics from the individual channel ROC with area under the curve (circle) and TPR at 5% FPR (square) metrics for data sampled at different rates. Analysis indicates improved performance with increasing sample rate, but with limited improvement above ∼0.6  Hz.

fNIRS devices often require a trade-off between the number of channels and the acquisition sample rate, and understanding the effect of this trade-off is of practical concern when designing auditory experiments; performance generally decreases with lower sample rates (Fig. 6). Analysis of data with a higher sample rate requires more memory and computational resources, so we selected 0.6 Hz as a sample rate that balances computational cost with accuracy.

#### 3.2.3 Effect of boxcar duration on the detection of auditory responses

**Fig. 7:**
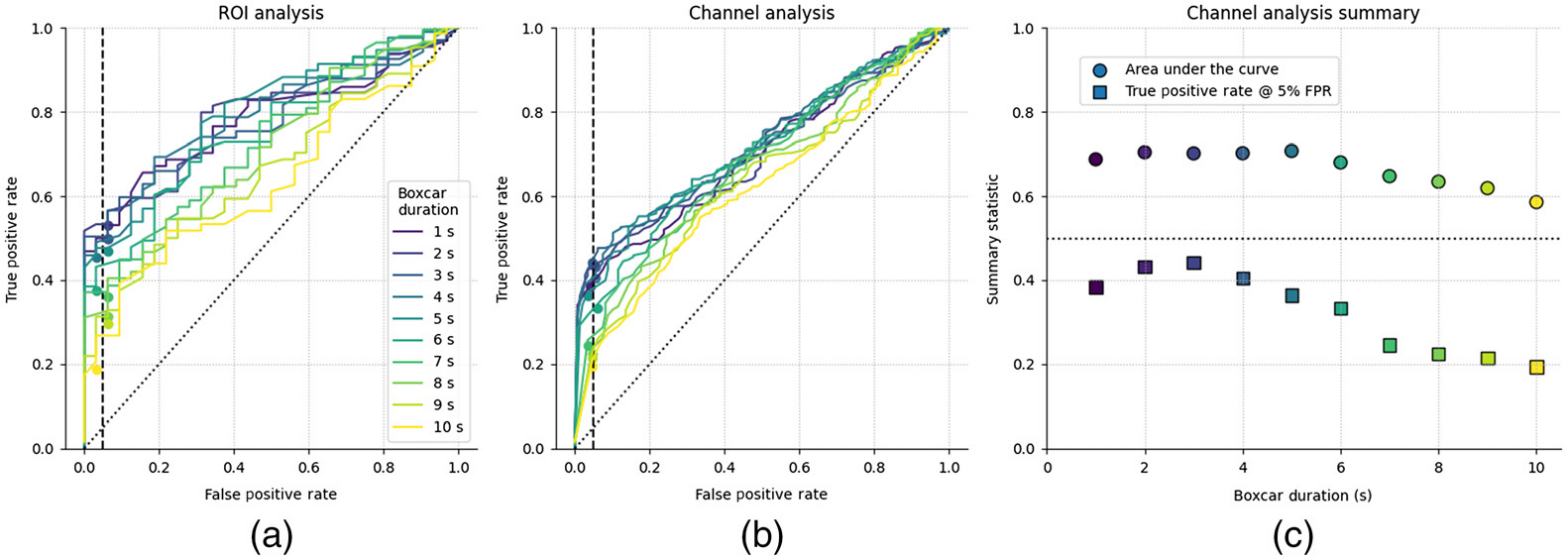
The effect of boxcar function duration on auditory fNIRS response estimates. (a) ROC curves for the STG region of interest and (b) individual channels over the STG. (c) Summary statistics from the individual channel ROC with area under the curve (circle) and TPR at 5% FPR (square) metrics for different boxcar durations. Analysis indicates optimal detection rates for a 3 s boxcar function; note that the stimulus duration was 5 s.

The fNIRS response to our 5 s block stimuli peaks around 6 to 7 s after stimulus onset (Fig. 4). GLM analyses fit an expected neural response to the data, in which the expected neural response is generated by convolving a model HRF with a boxcar function generated from the onset times of the stimuli. The length of the boxcar function can be varied to account for the duration of the neural response, and it is typically set to the duration of the stimulus. However, response morphology can change with stimuli and brain location. As such, we investigated the effect of boxcar length on response detection to auditory stimuli, and we find that the 3 s boxcar function provides the greatest TPR for a pre-determined 5% FPR (Fig. 7). Note, however, that the reduction in performance that comes from swapping out the 3 s boxcar function for one of 1 or 5 s duration is smaller than the reduction in performance that results from not employing systemic correction or when too low a sample rate is used. An alternative approach to account for differences between the model and the measured response is to include a derivative term in the design matrix.^13^^,^^75^ However, since we observed good correspondence between the response morphology and the expected canonical response, we did not include derivative terms in our analysis. The response morphology, and appropriate boxcar length, may vary with stimulus duration, characteristics, and brain structure of interest.^70^^,^^71^

Additional analysis parameters beyond the scope of the current study include effects arising from selection of the specific auto-regressive model^37^ or alternate canonical functions.^76^ Based on the data thus far, we maintained a sample rate of 0.6 Hz in future analyses and included all PCs of the short channels as regressors, employing a 3 s boxcar function to model the hemodynamic response.

### 3.3 Comparison of Conditions and Response Lateralization

Finally, we investigated whether, when applied at a group level, the averaging and GLM approaches to fNIRS analysis provide the same experimental conclusions. Two common questions in auditory experiments were explored. First, could we detect a difference in response amplitude between two conditions, in this example: speech and noise? Second, within one condition, is a difference in response amplitudes apparent across brain hemispheres, often termed “lateralization of responses”?

**Fig. 8:**
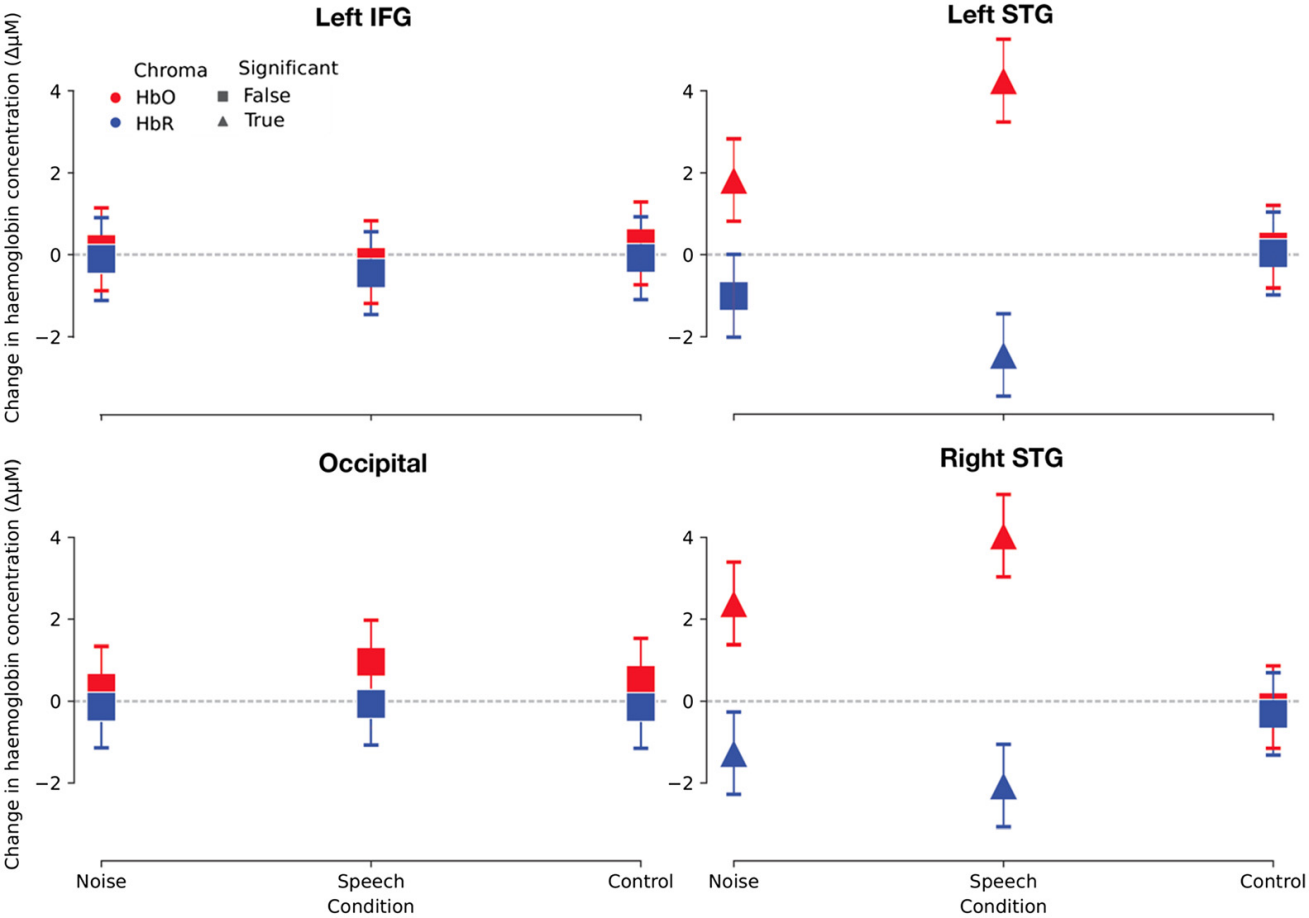
Estimates of response per condition and region of interest using the GLM analysis. Oxy- and deoxyhemoglobin responses are shown in red and blue, respectively. The presence of a response (statistical difference to zero) is indicated by a triangle. Error bars represent the 95% confidence intervals of the mean.

We first summarized the dataset (GLM analog of Fig. 3) by modelling the response amplitude as a factor of ROI, condition, and chromophore in a LME model, with the participant as a random factor (Fig. 8). Consistent with the observed average waveforms (Fig. 3), no significant responses were observed in either the left IFG or occipital lobe, and the silent, control condition generated no responses in any ROI. Significant responses were observed to both speech and noise in the two ROIs of the STG. The lack of any detectable response to speech stimuli in the left IFG may be due to the passive nature of the experimental task; this cortical region has been indicated in the processing of speech, particularly in active tasks with more challenging acoustic conditions.^9^^,^^14^

#### 3.3.1 Does speech elicit a greater neural response than noise?

We next addressed the questions of whether responses to speech are larger than responses to noise over the STG ROI and whether inter-hemispheric differences in activation are observed.

##### Comparison of averaging and GLM result

Using the Systemic Corrected GLM with a LME model, which examined the effect of condition with participant as a random effect, we observed that the speech-evoked oxyhemoglobin response was 2.043  μM larger than that evoked by noise (p<.001) and the speech-evoked deoxyhemoglobin response was −1.1  μM larger than that evoked by noise (p<0.001). Using the average waveform amplitude 5 to 7 s post stimulus onset, we observed that the estimated oxyhemoglobin response to speech was 1  μM larger than to the noise (p<.01) and the deoxyhemoglobin response to speech was −0.45  μM larger than to the noise (p<.01). From this, we conclude that both analysis methods generate the same experimental conclusion, consistent with visual inspection of the averaging and FIR GLM analyses (Fig. 3). The estimated response amplitude difference was larger for the GLM approach, possibly due to this approach better accounting for the statistical nature of the fNIRS noise.^37^ The time window used in the averaging approach may also reduce the estimated response amplitude, whereas a peak picking approach may result in a slightly larger estimate of the response. However, automated peak-picking approaches are prone to error, particularly when the signal-to-noise ratio is low, while manual methods of peak-picking reduce the repeatability of an analysis.

##### Effect of systemic component rejection

Analyzing the data using the GLM approach, with no correction for systemic responses—the No Correction analysis—indicates that the speech response was 2.306  μM larger than that to the noise stimulus (p=.025). Not including corrections for systemic responses generated a similar effect size to the Systemic Corrected analysis. This correspondence between methods of analysis may be due to the systemic response being relatively small or the systemic response being similar across conditions. Our experiment was a passive listening-task, and participants were asked not to pay attention to the stimuli. Studies that have observed an event-locked systemic component to auditory stimuli required participants to generate a response, for example, by means of a button press.^48^ These more active experimental paradigms may generate a larger systemic component and therefore elicit greater differences between analyses corrected or uncorrected for systemic effects. This differs from the individual-level findings in Sec. 3.2.1 in which the detection rate obtained from single channels increased when short channel systemic correction was applied. This suggests that inclusion of short channels for GLM analysis of passive auditory tasks is more important for studies investigating individual-participant effects rather than studies investigating group-level effects.

#### 3.3.2 Does speech elicit a larger response in left or right hemisphere?

##### Comparison of averaging and GLM result

Finally, to address whether a difference in response amplitude exists between the left and right cortical hemispheres to speech stimuli, results from the Systemic Corrected GLM were used in an LME model examining the effect of ROI, with participant as a random effect. The model reported that the estimated amplitude of the oxyhemoglobin fNIRS response in the right hemisphere was not significantly different from that in the left (β=−0.21, p=0.73), nor was there a difference for the deoxyhemoglobin (β=0.38, p=0.36). Similarly, the same LME model reported no significant lateralization of the oxyhemoglobin response amplitude across hemispheres when the averaging analysis was employed (β=1, p=.13), and similarly, the deoxyhemglobin showed no significant effect of hemisphere (β=−0.33, p=0.15).

##### Effect of systemic-component rejection

When assessing the No Correction GLM data at a group level, no significant effect of lateralization was observed (β=0.18, p=0.87), indicating that not compensating for systemic components does not generate aberrant lateralization effects. However, we cannot conclude from these data that, if a lateralization effect were present, it would be detectable without systemic correction.

#### 3.3.3 Discussion

Despite the limitations of the averaging approach compared with the GLM,^37^^,^^38^ both analyses resulted in the same experimental conclusion when applied to this dataset. When applied to both the comparison of conditions (speech versus noise) and comparison of cortical hemispheres (left versus right) research questions, the two analyses both resulted in the same conclusions. Thus, in the case of passive auditory experiments, meaningful comparisons can be made across studies applying either a GLM or averaging analysis.

## 4 Conclusion

A reference block-design auditory fNIRS dataset was created with two common acoustic stimuli. Using this dataset, it was determined that both an averaging approach and an FIR GLM analysis resulted in similar response morphology. The effect of correcting for systemic hemodynamic responses using short optical channels was evaluated on the response detection of the GLM approach; it was determined that including the individual short channels, or the PCs of the short channels, resulted in similar practical improvements in detection. At a group level, it was observed that both the averaging and GLM approaches produced the same experimental conclusions to two common research questions. Not including short-channel corrections did not change the group-level conclusions, which may be due to the task being passive in nature and may not hold for experiments requiring active participation. We conclude that, when appropriate averaging or GLM approaches are applied to passive auditory group-level data, both analysis pipelines generate similar results. This suggests that meaningful comparisons can be made across research groups that use different analysis approaches.

## Code, Data, and Materials Availability

The fNIRS data reported in this article will be released on OSF.io and github.com in the BIDS data format to allow for ease of reuse.^78^ All code functions used in this analysis are available at mne.tools/mne-nirs and the associated GitHub page, along with example analysis tutorials.
